# Is It Worth Performing Intersphincteric Resection in Patients Having Rectal Adenocarcinoma with Oligometastasis

**DOI:** 10.1007/s13193-024-02117-3

**Published:** 2024-10-26

**Authors:** Abdeali Saif Arif Kaderi, Sanjay Singh, Ankit Sharma, Mufaddal Kazi, Ashwin Desouza, Avanish Saklani

**Affiliations:** Department of Colorectal and Robotic Surgery, Department of Surgical Oncology, Tata Memorial Hospital, Homi Bhabha National Institute, Parel, Mumbai, Maharashtra India

**Keywords:** Intersphincteric resection, Oligometastatic ISR, Stoma non-reversal, Permanent stoma

## Abstract

Intersphincteric resection (ISR) is being increasingly performed in metastatic rectal adenocarcinoma (with oligometastasis) patients. There has been a trend towards worse prognosis in this group. This study compares the oncological and surgical outcomes of patients with and without pre-operative oligometastasis who underwent ISR. The outcomes compared include prognostic factors like margin positivity, recurrence rates, stoma reversal rate, and surgical failure rate (defined as a combination of one or more of the aforestated factors). The demographic pattern, American Society of Anaesthesia grade (ASA), treatment received, clinical and histopathological T and N stage, grade, type of minimally invasive surgery (MIS) approach, neoadjuvant therapy, and pathological high-risk features are also studied. Patients who underwent minimally invasive ISR over 10 years at a high-volume tertiary cancer center were selected for the study. Factors used for the assessment of oncological outcomes were margin positivity (circumferential resection and distal margin), recurrence (both local and systemic), and stoma reversal rate. A descriptive and comparative analyses were performed. Four hundred and eight patients underwent minimally invasive ISR of which 25 (6.12%) patients were oligometastatic. While R0 resection was similar in both groups, higher overall recurrence (24% versus 18.5%) and low stoma reversal rate (36.8% versus 67.3%) were observed in the oligometastatic group with statistically significant differences. No local recurrence was seen in the oligometastatic group, and the difference compared to non-metastatic group was not statistically different. This study indicates that oligometastatic patients who underwent ISR experienced lower stoma reversal rates, maintaining similar local control but facing higher rates of systemic recurrence.

**Supplementary Information:**

The online version contains supplementary material available at 10.1007/s13193-024-02117-3.

## Introduction

Intersphincteric resection (ISR) [1] is a well-established sphincter saving procedure for low rectal cancers where intersphincteric space is free and not suitable for stapled anastomosis. While the outcome of locally advanced rectal cancers depends on local recurrence, and disease-free survival (DFS), stoma reversal is an important endpoint of sphincter-saving procedure. Fifteen to 20% of ISR/low anterior resection (LAR) do not undergo reversal due to anastomotic complications or distant metastasis despite locoregional control. This situation gets more complicated in oligometastatic disease, who undergo either synchronous or metachronous resections [2–6]. At what point stoma reversal would fit in between these procedures is difficult. While the oncological outcomes of metastatic diseases have validated the performance of major resections in landmark series [7], the quality of life needs to be given equal if not more importance, due to the improved survival. However, permanent stoma avoidance, which was the necessity for this invention, itself became its Achilles heel.

The aim of this study is to study the oncological and surgical outcomes of patient undergoing ISR who had oligometastatic disease with non-metastatic cases. A composite end point viz. surgical failure was defined as margin positivity rate, recurrence rate, and stoma reversal rate, or a combination of these was evaluated. Functional sphincter preservation is pivotal in the surgical outcomes of ultra-low anterior resection and ISR. A significant proportion of patients undergoing ISR have incontinence [8] resulting in technically permanent diversion stomas [9]. Similarly, patients with intact sphincters and early recurrences (3.4%) [9] lose the chance of stoma reversal. We studied the oncological outcomes in oligometastatic low rectal cancers who underwent ISR to compare the outcomes with non-metastatic counterparts and appraise the futility of performing ISR in metastatic cases.

## Methods

### Patients, Setting, and Design

This is a single institution retrospective study. Consecutive patients who underwent minimally invasive (MIS) and open ISR over the period of January 2013to March 2024 at a high-volume tertiary cancer center were selected for the study. Patients of all age groups and metastases status were included.

### Variables and Outcomes

Electronic medical records of these patients were reviewed and prognostic factors like margin positivity (circumferential resection and distal margin), recurrence (both local and systemic) and stoma reversal rate were noted. Surgical failure was defined as any or a combination of positive margin, local recurrence, and stoma non-reversal rates. The demographic pattern, American Society of Anaesthesia grade (ASA), treatment received, clinical and histopathological T and N stage, grade, type of MIS approach, neoadjuvant therapy, and pathological high-risk features were recorded.

### Statistics

Continuous variables were represented by medians and interquartile range and group comparisons were performed using the Mann–Whitney *U* test. Similarly, categorical variables were represented by numbers and proportions, while comparisons were performed using the chi-squared test. A *p* value of < 0.05 was considered of significance. All statistical analyses were conducted using Microsoft Office Excel version 2019 and IBM SPSS version 25 (IBM Co., Armonk, NY, USA).

### Ethics

The study protocol was designed and conducted in accordance with the ethical standards of the institutional research committee and the 1964 Helsinki Declaration and its later amendments. Because the study was retrospective and used anonymized data without patient contact, the institutional research board waived the need for approval. The study adhered to the Strengthening the Reporting of Observational Studies in Epidemiology (STROBE) guidelines.

## Results

Four hundred and sixteen patients underwent minimally invasive ISR. Out of these, metastatic versus non-metastatic information was not available for 8 patients. Thus, the final analysis was performed on four hundred and eight patients. The age, sex ratio, ASA grade, and body mass index (BMI) were comparable in both groups. Similarly, oncological factors like histological grades, clinical T and N stage, extramural vascular invasion (EMVI), and type of MIS approach were comparable. The surgical outcomes viz. circumferential resection margins (CRM) and distal recurrence margins (DRM) were comparable. Similarly, the oncological outcome viz. local recurrence was comparable. Twenty-five (6.12%) patients were having pre-operative oligometastasis, and the rest (93.88%) were non-metastatic. Consequently, the oligometastatic group had a higher proportion of patients receiving neoadjuvant chemotherapy. A higher recurrence of 24% (6 out of 25 patients) and a low stoma reversal rate of 36.8% were observed in the oligometastatic group with a statistically significant difference compared to 67.3% stoma reversal rate and a 18.5% (71 out of 383) recurrence rate in the non-metastatic group. Though no local recurrence was seen in the oligometastatic group, the difference was not statistically different. However, the overall recurrence was higher in the oligometastatic group (24%% versus 18.5%) with a *p* value of 0.499. Finally, surgical failure was seen in 148 patients (38.7%)% (4% CRM positive, 2.9% distal margin positive, 11.5% pelvic recurrence, and 32.7% stoma non-reversal rate) in the non-metastatic group and 13 patients (52%) (5% CRM positive and 63.2% stoma non-reversal rate) in the oligometastatic group (*p* 0.009). The demographic factors and the outcome factors are summarized in Table 1.

**Table 1:** Demographic and outcome factors comparing 408 patients non-metastatic and metastatic patients undergoing intersphincteric resection (ISR) over 11 years

		Metastases			
Variable	Categories	Without pre-operative metastasis (383)	With pre-operative oligometastasis (25)	Total	*p* value
Age	Median (IQR)	47 (37–56)	45 (40–56)	47 (37–56)	0.999
Pre-operative albumin	Median (IQR)	4.1 (3.8–4.3)	4 (3.49–4.095)	4.07 (3.8–4.3)	0.017
Body mass index (BMI)	Median (IQR)	23.835 (21.575–25.9625)	23.27 (20.5–25.4)	23.8 (21.5–25.93)	0.547
Serum CEA	Median (IQR)	3.4 (2–6.3)	4.6 (2.75–10)	3.4 (2.07–6.5)	0.034
Surgery duration	Median (IQR)	300 (250–372.5)	350 (240–450)	300 (245–377.5)	0.175
Blood loss	Median (IQR)	250 (200–400)	400 (325–1100)	250 (200–400)	< 0.001
Sex	Female	112 (29.2)	11 (44)	123 (30.1)	0.119
	Male	271 (70.8)	14 (56)	285 (69.9)	
Total		383 (100)	25 (100)	408 (100)	
ECOG performance status	0	4 (1)	0 (0)	4 (1)	0.681
	1	323 (84.3)	20 (80)	343 (84.1)	
	2	56 (14.6)	5 (20)	61 (15)	
Total		383 (100)	25 (100)	408 (100)	
ASA	1	219 (57.3)	15 (60)	234 (57.5)	0.820
	2	154 (40.3)	9 (36)	163 (40)	
	3	9 (2.4)	1 (4)	10 (2.5)	
Total		382 (100)	25 (100)	407 (100)	
Preoperative T stage	T1	5 (1.3)	0 (0)	5 (1.2)	0.087
	T2	73 (19.3)	6 (24)	79 (19.6)	
	T3	273 (72.2)	14 (56)	287 (71.2)	
	T4	25 (6.6)	4 (16)	29 (7.2)	
	Tis	2 (0.5)	1 (4)	3 (0.7)	
Total		378 (100)	25 (100)	403 (100)	
Preoperative N stage	N0	91 (23.9)	4 (16)	95 (23.5)	0.009
	N1	185 (48.7)	7 (28)	192 (47.4)	
	N2	104 (27.4)	14 (56)	118 (29.1)	
Total		380 (100)	25 (100)	405 (100)	
Neoadjuvant therapy	No	49 (12.9)	2 (8)	51 (12.6)	0.477
	Yes	332 (87.1)	23 (92)	355 (87.4)	
Total		381 (100)	25 (100)	406 (100)	
NACTRT	No	91 (23.9)	13 (52)	104 (25.7)	0.002
	Yes	289 (76.1)	12 (48)	301 (74.3)	
Total		380 (100)	25 (100)	405 (100)	
Surgery	ISD	4 (1)	2 (8)	6 (1.5)	0.019
	ISR	375 (98.4)	23 (92)	398 (98)	
	PISR	2 (0.5)	0 (0)	2 (0.5)	
Total		381 (100)	25 (100)	406 (100)	
LVI (lymphovascular invasion)	No	358 (95.5)	23 (92)	381 (95.3)	0.430
	Yes	17 (4.5)	2 (8)	19 (4.8)	
Total		375 (100)	25 (100)	400 (100)	
PNI (perineural invasion)	No	361 (96.3)	23 (92)	384 (96)	0.292
	Yes	14 (3.7)	2 (8)	16 (4)	
Total		375 (100)	25 (100)	400 (100)	
EMVI (extramural vascular invasion)	No	359 (95.7)	24 (96)	383 (95.8)	0.949
	Yes	16 (4.3)	1 (4)	17 (4.3)	
Total		375 (100)	25 (100)	400 (100)	
Number of positive nodes	Nodes negative	273 (73.6)	12 (50)	285 (72.2)	0.012
	Nodes positive	98 (26.4)	12 (50)	110 (27.8)	
Total		371 (100)	24 (100)	395 (100)	
Circumferential resection margin	Negative	361 (96)	24 (96)	385 (96)	0.998
	Positive	15 (4)	1 (4)	16 (4)	
Total		376 (100)	25 (100)	401 (100)	
Distal margin	CRM	23 (6)	1 (4)	24 (5.9)	0.625
	No	349 (91.1)	24 (96)	373 (91.4)	
	Yes	11 (2.9)	0 (0)	11 (2.7)	
Total		383 (100)	25 (100)	408 (100)	
Peritoneal recurrence	No	196 (93.8)	12 (85.7)	208 (93.3)	0.243
	Yes	13 (6.2)	2 (14.3)	15 (6.7)	
Total		209 (100)	14 (100)	223 (100)	
Pelvic recurrence	No	184 (88.5)	14 (100)	198 (89.2)	0.178
	Yes	24 (11.5)	0 (0)	24 (10.8)	
Total		208 (100)	14 (100)	222 (100)	
Recurrence in liver	No	193 (92.8)	8 (57.1)	201 (90.5)	< 0.001
	Yes	15 (7.2)	6 (42.9)	21 (9.5)	
Total		208 (100)	14 (100)	222 (100)	
Recurrence in lung	No	193 (92.8)	12 (85.7)	205 (92.3)	0.335
	Yes	15 (7.2)	2 (14.3)	17 (7.7)	
Total		208 (100)	14 (100)	222 (100)	
Recurrence in retroperitoneal nodes	No	193 (91.9)	14 (100)	207 (92.4)	0.268
	Yes	17 (8.1)	0 (0)	17 (7.6)	
Total		210 (100)	14 (100)	224 (100)	
Anastomosis stricture	No	220 (80.3)	15 (93.8)	235 (81)	0.182
	Yes	54 (19.7)	1 (6.3)	55 (19)	
Total		274 (100)	16 (100)	290 (100)	
Anal dilatation	No	154 (79.8)	10 (90.9)	164 (80.4)	0.366
	Yes	39 (20.2)	1 (9.1)	40 (19.6)	
Total		193 (100)	11 (100)	204 (100)	
Stoma reversal	No	116 (32.7)	12 (63.2)	128	0.006
	Yes	355 (67.3)	7 (36.8)	362	

The oligometastatic cohort included 14 patients with liver metastasis, 3 patients with both liver and lung metastases, 1 patient with liver, lung, and peritoneal metastases, 4 patients with paraaortic lymph node metastases, 1 patient with both paraaortic lymph node and lung metastases, and 2 patients with only lung metastases. Table 2 provides a summary of the treatments administered and their outcomes.

## Discussion

Our study aimed to understand the percentage of patients with oligometastatic disease who underwent ISR. Furthermore, we analyzed what percentage of these patients underwent stoma reversal and compared that with the non-metastatic group. Oligometastatic disease needs additional adjuvant chemotherapy and ancillary procedures. Thus, the time point suitable for stoma reversal is a logistic concern. On average, the adjuvant chemotherapy starts 3–4 weeks after the surgery and lasts for 6–9 months. Whether stoma closure should be done during the chemotherapy or after completion of chemotherapy is a matter of debate and personal preference.

ISR offers a combination of surgical as well as well as oncological outcomes. Our series has a local recurrence of 6.1% in the non-metastatic group and none in the oligometastatic group in congruence with the contemporary groups [10, 11]. The margin positivity rate was 3% in non-metastatic group and nil in oligometastatic group. This is comparable to close margin rates in the contemporary series [10] of 3.2%. In our previously published series of 310 patients undergoing ISR, the surgical failure was seen in 21% of patients. Out of these, positive margin was seen in 3.5%, local recurrence in 5.8%, and an unreversed stoma in 17%. A higher number (*n* = 12, 12%) of systemic recurrences were seen in the preoperative M1 patients [12]. In another series of 142 patients published by our group, comprising of poorly differentiated and signet ring cell tumors(PDSR), local recurrence was seen in 15.6% in the PDSR group and 11.7% in the non-PDSR group, and the difference was not significant [13]. Only tumor stage and not the histology was a predictor of recurrence.

The treatment algorithms have been reviewed time and again, and the treatment sequence is based on the whether the primary is symptomatic and metastasis is resectable. A large series of 92 patients of CRLM from our institute has 22 patients undergoing LAR/ISR as a part of simultaneous resection. Although it demonstrated feasibility of simultaneous proctectomy with liver resection, rectal carcinomas, especially undergoing ISR, have been under-represented in most of the series [14]. None of the series explore this aspect of treatment planning in rectal carcinoma with oligometastases to the best of our knowledge.

Having a stoma significantly affects day to day life of patients and some show reluctance to even temporary stomas to the extent that they refuse curative surgery. Social embarrassment and body image are major issues faced by patients with stoma. To avoid such predicament, a proper counselling of stoma care is of utmost importance [15].

Older age, ASA score > 2, comorbidities, open surgery, surgical complications, anastomotic leakage, stage IV tumor, and local recurrence have been reported as risk factors for non-closure of defunctioning stomas after anal preservation surgery in one meta-analysis [16]. Patients with these risk factors need to be informed preoperatively of the possibility of non-reversal [17–19]. A retrospective study from South Korea reported tumor progression with local and systemic recurrence as a cause of permanent stoma [20]. This study recognized the relevance of metastatic status. It was found that metastatic cases had higher chances of permanent stoma (hazard ratio (HR), 3.380; 95% confidence interval (CI), 1.192–18.023; *p* = 0.027). In a retrospective study [21] of low anterior resection, 12% of stoma were not reversible, 11% required a repeat stoma after stoma closure, and 10% received a late permanent stoma. The risk factor identified were male gender, low tumor site, advanced stage, and anastomotic complications. Despite the reduction in recurrences over the past decade, the rate of permanent stoma did not change. One center from Seoul [22] reported anastomotic complication (7 out of 71 permanent stoma) to be the primary cause of early permanent stoma and recurrence (27 out of 71 permanent stoma) to be the cause of late permanent stoma. Yet, another group [23] from Korea reported a series of 2528 patients who underwent surgery for low rectal cancers and had 28 out of 231 patients having permanent stoma due to either anastomotic leak or recurrence. Thus, metastatic status has been less recognized as a risk factor for stoma non-reversal. These studies have very small number of metastatic Ca rectum who underwent ISR and thus cannot identify all the predictors of successful reversal. A higher number of cases of this subgroup is needed to conclusively prove the same.

Based on these studies, it can be inferred that patients undergoing ISR for metastatic rectal cancer have a lower likelihood of being stoma-free. Therefore, patient selection for ISR in a metastatic setting must be more stringent. While there is no definitive evidence to completely rule out ISR in metastatic cases, it is evident that oligometastatic patients are prone to distant failures due to the inherent nature of the disease.

Patients with recurrence often request sphincter-saving procedures, but ISR is associated with functional disabilities and requires approximately a year of rehabilitation. In metastatic cases, there is a higher likelihood of ongoing chemotherapy and increased systemic relapse rates. Our study indicates that the opportunity for stoma closure in these patients is limited, with 63.2% likely to end up with a permanent ileostomy. For this cohort, a shorter surgery such as abdominoperineal resection (APR) or intersphincteric APR might be a better option.

Our data can be used to counsel patients with locally advanced cancers and oligometastatic disease who insist on ISR after neoadjuvant therapy about the high chances of non-reversal of stoma.

Our study is not without limitations. It is a single-center retrospective study. The surgical failure was assessed in terms of local recurrence, persistent stoma rates, and margin positivity. However, functional outcomes in terms of sphincter tone, incontinence rates, and low anterior resection scores were not recorded. Furthermore, ours is a group of robotic and laparoscopic ISR; hence, the effects are blended and the heterogeneity in terms of surgical failure caused by adopting either approach is not well demarcated. A short-term follow-up of the recently operated group is another limitation; hence, part of our data is in the preliminary phase. Follow-up is going on and is expected to give mature results.

## Conclusion

This study shows that the patients who undergo ISR with pre-operative oligometastasis experience higher recurrence and low stoma reversal rates without affecting margin positivity. It is a prerequisite to carefully counsel the patient as regards the chances of a permanent stoma in such scenarios. The patients’ expectations may not be met if the early disease recurrence hampers stoma reversal in oligometastatic cases. Hence, higher than usual chances of a stoma non-reversal must be acceded by the patient while consenting for this procedure. Since the number of patients with oligometastasis is very low in the present study, a larger sample size is required to translate this conjecture into evidence.

## Supplementary Information

Below is the link to the electronic supplementary material.

Supplementary file1 (DOCX 18 KB)

## Data Availability

This data used in this publication is stored in the parent institute in the electronic medical records and shall be made available on appropriate requests.
