# Supplementary material for: Is It Worth Performing Intersphincteric Resection in Patients Having Rectal Adenocarcinoma with Oligometastasis
Source: Indian J Surg Oncol. 2024 Oct 26;16(2):645–50. doi: 10.1007/s13193-024-02117-3 (PMC12052657; doi:10.1007/s13193-024-02117-3)
Supplement: Supplementary file 1 — Supplementary file1 (DOCX 18 KB) [file 13193_2024_2117_MOESM1_ESM.docx]

| Supplimentary TableSr No | Sites of metastases | Treatment Modality | Treatment response | Recurrence | Alive/Dead | Comments |
| --- | --- | --- | --- | --- | --- | --- |
| 1 | Liver | TACE | Good response | No | Death due to other cause |  |
| 2 | Liver | DEBIRI-TACE | Good response | Recurrence🡪 SBRT🡪 Recurrence | Death | Progressive disease after second recurrence |
| 3 | Liver | RFA | Good response | No |  | Loss to follow up |
| 4 | Liver | Metastectomy +TACE | Good response | No | Alive |  |
| 5 | Liver | DEBIRI-TACE | Good response | Yes🡪 PD | Death |  |
| 6 | Liver | RFA | Good response | No | Alive |  |
| 7 | Liver | RFA | Good response | Yes🡪 DEBIRI-TACE | Alive |  |
| 8 | Liver | RFA | Good response | No | Alive |  |
| 9 | Muliple Liver Mets | NACT | Good response (Vanished) | Yes | Death |  |
| 10 | Single Liver met | NACT +Bevacizumab | Good response | Yes |  | Loss to follow up |
| 11 | Single Liver met | NACT | Partial response | Planned for resection | Death | Progressive disease in the interim period. |
| 12 | Single liver met | NACT | Good response  (Vanished) | No | Alive |  |
| 13 | Lung +Liver met | NACT | Good response  (Vanished) | Yes | Death |  |
| 14 | Liver+Multiple lung met | NACT | Partial response | Progressive disease with further lines of chemotherapy | Death |  |
| 15 | Lung | NACT | Partial response | Residual disease🡪CTRT | Alive |  |
| 16 | Paraaortic lymph nodes | NACT | Good response | No | Alive |  |
| 17 | Paraaortic lymph nodes | NACT🡪 Surgery | Good response | No | Alive |  |
| 18 | Paraaortic lymph nodes | Radiation to the PALN | Good response | Peritoneal +Pelvic recurrence🡪 CRS HIPEC | Alive |  |
| 19 | Lung+ Paraaortic nodes | NACT | Good response | Lung lesion responded, Paraaortic node operated. | Alive |  |
| 20 | Lung | NACT 🡪 Metastectomy | Good response | No | Alive |  |
| 21 | Liver + lung | NACT | Good response in liver. Metastectomy done for lung | No | Alive |  |
| 22 | Liver | RFA🡪 HAIC | Good response  (Vanished) | Recurrence in lung🡪 metastectomy | Alive |  |
| 23 | Liver | RFA | Good response | No | Alive |  |
| 24 | Liver+ lung+ Peritoneum | NACT🡪CRS | Good response | No | Death |  |
| 25 | Paraaortic lymph node | NACT 🡪 Surgery |  | No | Alive |  |

Table 2: Sites of Oligometastases and the treatment and outcomes
